# Mobile robots exploration through cnn-based reinforcement learning

**DOI:** 10.1186/s40638-016-0055-x

**Published:** 2016-12-21

**Authors:** Lei Tai, Ming Liu

**Affiliations:** 1 Department of Mechanical and Biomedical Engineering, City University of Hong Kong, Tat Chee Avenue, Kowloon Tong, 999077 Hong Kong; 2 Department of Electronic and Computer Engineering, HKUST, Clear Water Bay, Kowloon, 999077 Hong Kong

**Keywords:** Q-learning, Deep learning, Robot exploration

## Abstract

Exploration in an unknown environment is an elemental application for mobile robots. In this paper, we outlined a reinforcement learning method aiming for solving the exploration problem in a corridor environment. The learning model took the depth image from an RGB-D sensor as the only input. The feature representation of the depth image was extracted through a pre-trained convolutional-neural-networks model. Based on the recent success of deep Q-network on artificial intelligence, the robot controller achieved the exploration and obstacle avoidance abilities in several different simulated environments. It is the first time that the reinforcement learning is used to build an exploration strategy for mobile robots through raw sensor information.

## Background

 For mobile robots, exploration in an unknown environment is always a fundamental problem in various areas, such as rescue and mining. Typically, robot requires complicated logic about the obstacles and the topological mapping of environments [1, 2] designed by human beings based on the information provided from vision or depth sensors. It is still a challenge to achieve this task rapidly. And high-level human-brain-like intelligence is rarely considered in these traditional areas.

Recently, convolutional neural networks [3], also called deep learning, have attracted more and more attentions in artificial intelligence. This hierarchical model shows great potential in feature representations. Regarding the requirements mentioned above, deep reinforcement learning (DRL), merging reinforcement learning and deep learning, is a proper method to apply in this scenario. For example, Google DeepMind implemented a deep Q-network (DQN) [4] on 49 Atari-2600 games. This method outperformed almost all of other state-of-the-art AI controllers and 75% human players, without any prior knowledge about the Atari 2600 games. It showed great potential to apply this algorithm in other related fields including mobile robots exploration.

In this paper, we developed a CNN-based reinforcement learning method for mobile robots to explore an unknown environment based on raw sensor information. Not like the DQN mentioned above, we separated this learning approach in two separate networks, the perception and the control networks. Firstly, we built a supervised learning model as the perception network by taking the depth information as the input and the command of the robot as the output. The datum was manually labeled with control commands to tune the moving directions of the mobile robot. This supervised learning model was implemented as three convolutional layers. Secondly, the control network was constructed with three fully connected hidden layers to mimic the Q-value approximation of the reinforcement learning procedure by taking the feature representations extracted by the perception networks as the input. The feature representations were the output of the last convolutional layer of the perception network trained before. This reinforcement learning framework was defined as a CNN-based reinforcement learning method. Particularly, we stressed the following contributions:

- We designed a revised version of DQN network for a mobile robot to explore an unknown environment only receiving the raw sensor information as the interaction with the environment.
- The model was validating in several simulated environments. The experiments in simulated environments reflected the effectiveness of the method.

The training and construction of the perception network were based on our previous work [5]. The preliminary experiment was described in [6]. The rest of paper is organized as follows: We present related works in reinforcement learning and CNN perception in Related work section. In Implementation details section, we describe implementation about the origin DQN and our CNN-based reinforcement learning method. The detail of the training and tests is then presented in Experiments and results section. At the end, Conclusion section concludes the paper and introduces the future work.

## Related work

### Reinforcement learning in robotics

Reinforcement learning (RL) [7] is an efficient method for robotics to interact with the environment and to learn skills by self-motivation. With an appropriate and abstract reward, the robot can learn a complex strategy without ground truth labeled by human beings as references. It was just applied on mastering the strategy of GO (an ancient Chinese board game which was regarded as the most challenging task for artificial intelligence) [8] and overcame the best human GO player. It indicated the great feasibility of reinforcement learning in other fields. RL was also applied on an autonomous helicopter flight [9] and autonomous inverted helicopter flight [10], by collecting the flight data and learning a nonlinear model of the aerodynamics.

Reinforcement learning was also proved to improve the motion behavior of a humanoid robot to react to visually identified objects substantially [11], by building an autonomous strategy with little prior knowledge. In this application, the robot showed a continuously evolved performance with time.

Most of the reinforcement learning methods for robotics were based on state information like joint states of robot arms. To our knowledge, raw image sensor information has never been considered directly.

### CNN in perception

Convolutional neural network (CNN) is a classic visual learning method. With the development of large-scale computing and GPU accelerating, huge CNN frameworks can be set with tens of convolutional layers. The newest development of residual network [12] applied in image classification even used more than 150 convolutional layers.

Normally, CNN was used to solve a classification problem with a softmax layer, such as Imagenet classification [13, 14] and face recognition [3]. With a regression layer to optimal the Euclidean loss, the feature maps extracted by CNN can also be applied to key points searching problem [15, 16]. Computer-vision-based recognition methods are mainly feature detection and extraction [17–19], while CNN extracts this feature model by self-learning.

In terms of robotics, CNN was also used to perceive environment information for visual navigation [20]. However, a supervised-learning-based method requires a complicated and time-consuming training period and the trained model cannot be applied in a different environment directly.

## Methods

Traveling in an unknown environment with obstacle avoidance ability is the main target of this paper. This task was defined as controlling a ground-moving robot in an environment without any collisions with the obstacles. For the perception network, CNN supervised learning model for the extraction of feature representations was trained in our previous work [5]. The training dataset of perception network was sampled from a corridor environment, through teleoperation from a human being as labeling. Based on this perception model, the robot can navigate without collision automatically in the similar real-world corridor environment. The supervised learning model is quite appropriate to perceive an environment [20]. And the experimental results in [5] proved that the feature representations can be effectively regarded as abstracted information for the environment to some extends. However, the serious overfitting of supervised learning model limited the extension of the trained model. To be rapidly adapted to some new environment, we proposed the control network so that robots can be adapted to some new environment rapidly. Based on the advantage of reinforcement learning, the extra labeling process for the new environment was also eliminated. The implementation of the experiment includes three parts:

- a simulated 3D environment in Gazebo for a robot to explore.
- a CNN-based reinforcement learning control framework.
- a simulated Turtlebot with a Kinect sensor in Gazebo controlled by the output of the proposed model.

### DQN

DQN defined the tasks between the agents and the environments [4, 21] in Atari 2600 games. The environment was set as $\varepsilon$. At each step, the agent selected an action $a_t$ from the action sets of the game and observed a displayed image $x_t$ from the current screen. The change in the game score $r_t$ was regarded as the reward for the action with the related state. For a standard reinforcement learning procedure, all of these game sequences $s_t$ were considered as a Markov decision process directly (MDP), where $s_t=x_1,a_1,x_2,\ldots ,a_{t-1},x_t$. Defining the discounted reward for the future by a factor $\gamma$, the sum of the future reward until the termination of the game would be $R_t=\sum _{t'=t}^{T} {\gamma }^{t'-t}r_{t'}$. *T* means the termination time step of the game. The target was to maximize the action-value function $Q^{*}(s,a) = \max _{\pi }{\mathbb {E}}[R_t|s_t=s,a_t=a,\pi ]$, where $\pi$ is the strategy for choosing of best action. From the Bellman equation, it is equal to maximize the expected value of $r+{\gamma }Q^{*}(s',a')$, if the optimal value $Q^{*}(s,a)$ of the sequence at the next time step is known as

$$\begin{aligned} Q^{*}(s ,a )={\mathbb {E}}_{{s'}\sim {\varepsilon }}\left[ r+\gamma \max \limits _{a'}Q^{*}(s',a')|s,a\right] \end{aligned}$$

Not using iterative updating method to optimal the equation, it is common to estimate the equation by using a function approximator. Q-network in DQN was such a neural network function approximator with weights $\theta$ and $Q(s,a,\theta ) \approx Q^{*}(s,a)$. Then, the real-time optimal Q-value for (*s*, *a*) can be estimated as

$$\begin{aligned} Q^{*}(s,a)={\mathbb {E}}_{{s'}\sim {\varepsilon }}\left[ r+\gamma \max \limits _{a'}Q^{*}(s',a';\theta _i)|s,a\right] \end{aligned}$$

The loss function to train the Q-network is

$$\begin{aligned} L_i({\theta }_i) = {\mathbb {E}}_{(s,a)\sim \rho (\cdot )}\left[ (y_i-Q(s,a;{\theta }_i))^2\right] \end{aligned}$$

where $y_i$ is the target and it is calculated by the equation for $Q^{*}(s,a)$ mentioned above based on reward and the estimation for next state $s'$. $\rho (\cdot )$ is the probability distribution of sequences *s* and *a*. By minimizing the loss between $y_i$ and *Q*(*s*, *a*), motivate the weights $\theta _i$ to converge. The gradient of the loss function is shown below:

$$\begin{aligned} \nabla _{\theta _{i}} L_{i}(\theta _{i}) = {\mathbb {E}}_{s,a \sim \rho (\cdot );s' \sim \varepsilon }\left[ (y_i-Q(s,a;\theta _i))\nabla _{\theta _i}Q(s,a;\theta _i)\right] \end{aligned}$$

### CNN-based reinforcement learning system

**Fig. 1:**
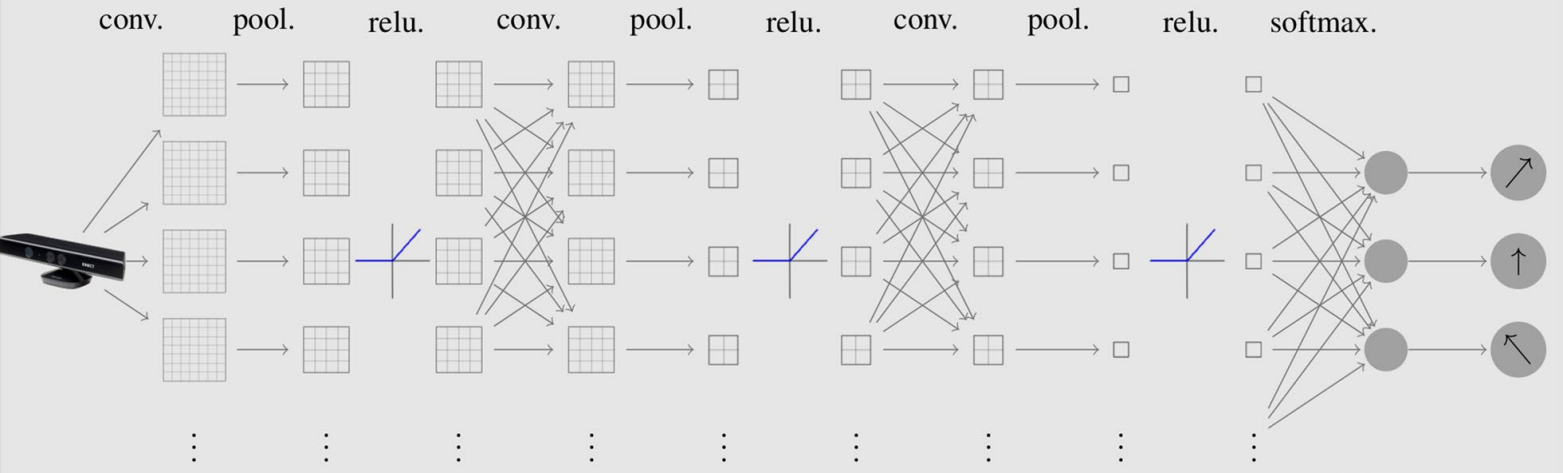
Structure of the perception network. Depth images after downsampling are fed into the model. Three convolutional layers with pooling and rectifier layers behind are connected together. After that, feature maps of every input are fully connected and fed to the softmax layer of the classifier

To accomplish the task of exploration, we simplified the DQN [4] into two separate networks, the perception network and the control network. For the perception network, a 3-layer CNN framework was built to do the preprocessing procedure. Figure 1 shows the CNN structure in detail. By three times convolution, pooling, and rectifier activation, the feature maps of the inputs were extracted. A softmax layer was implemented to get the output distribution of the moving commands. When training the network, we controlled the robot to explore in an environment and labeled the depth image from Kinect RGB-D sensor. The related control command from a human being was labeled as the ground truth. The parameters, training and analysis of this CNN model were introduced in our previous work [5] .

**Fig. 2:**
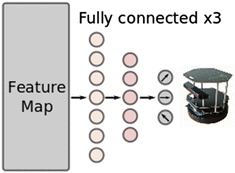
Feature maps extracted from the perception network are the input of the control network. They are reshaped to a one-dimensional vector. After three fully connected hidden layers of a neural network, it is transformed to the three commands for moving directions as the output

**Fig. 3:**
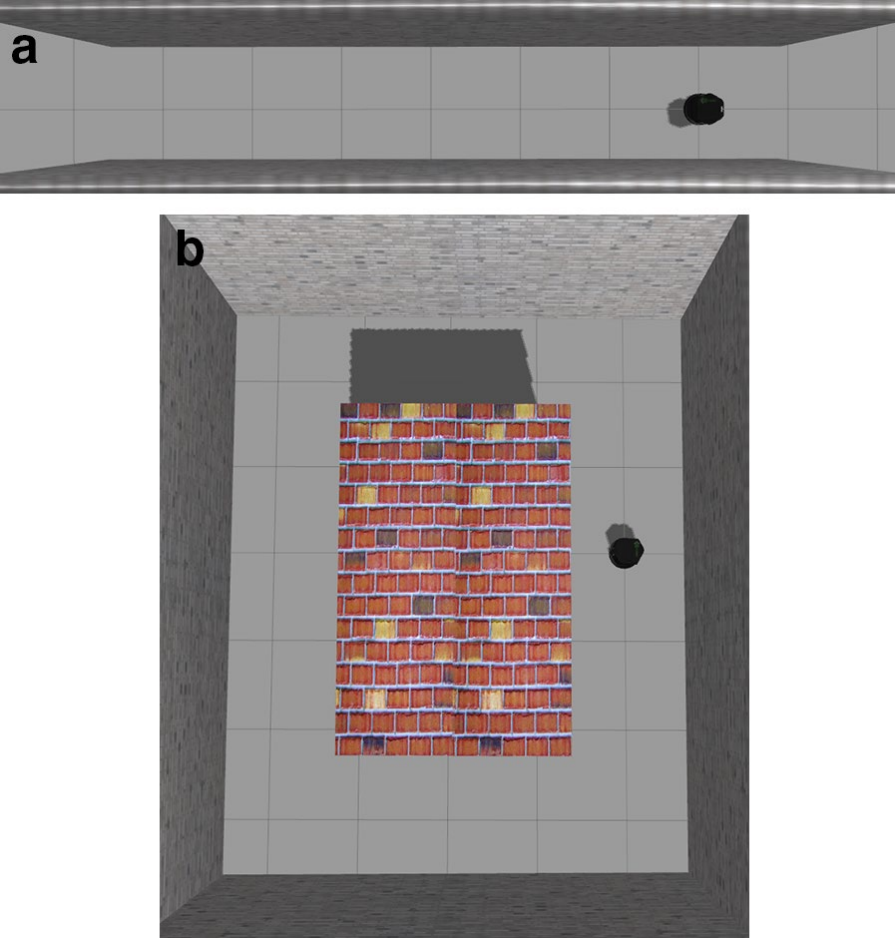
Simulation environments. **a** Straight corridor, **b** circular corridor

**Fig. 4:**
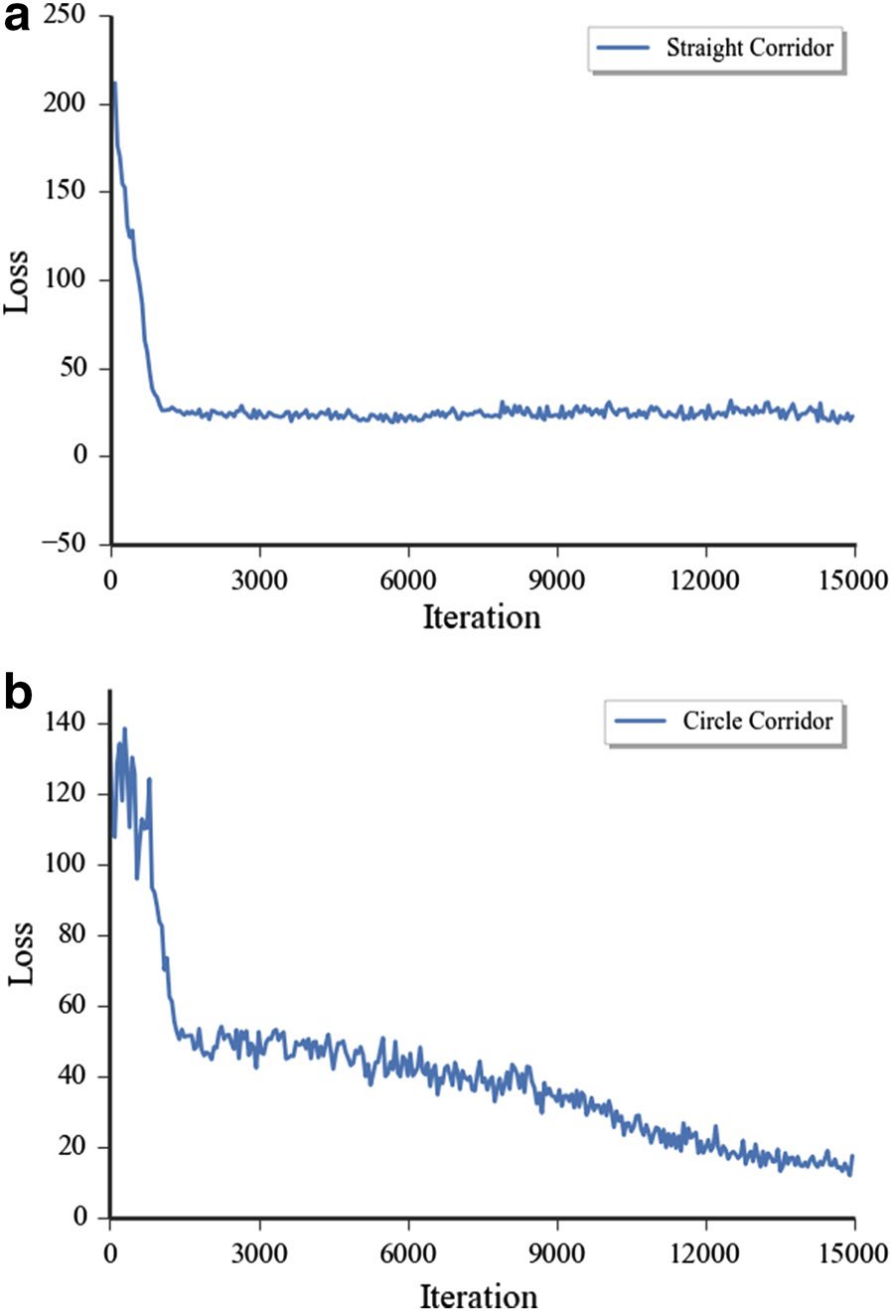
Converging curves of batch loss in iteration procedure. **a** Straight corridor training loss, **b** circular corridor training loss

In the control network, the reinforcement learning procedure, the trained model mentioned above was used to forward every input depth image of real time and get the feature maps of the depth image. Feature maps are the output of the last ReLU layer in Fig. 1. Note that we did not use the output command from the perception network directly. The feature representations consisted of $64\times 20\times 15$ matrices. Figure 2 shows the structure of the neural network to estimate the Q-value of the control network. The feature representations were regarded as the states $s_t$ as described in DQN section. In the exploration period, the depth image from Kinect was firstly processed by the pre-trained perception network to be the real-time state of the mobile robot. After the activation of the action, the new depth image is captured and process to be the next time state $s_{t+1}$. The real-time state $s_t$ is memorized with the related action $a_t$, reward *r* and the next state $s_{t+1}$ as a transition. As the same memory strategy in DQN, we reserved a replay memory to save the transition. At the same time, we randomly chose a batch of transitions to update the weights $\theta _i$ of the control network through stochastic gradient descent method.

The reward function for the control network had two different feedback values, one for normal moving and one for the collision with the obstacles. The system stared at the Turtlebot state by checking the minimum depth between the Turtlebot and the obstacles based on the sensor receiving from the Kinect. Table 2 shows the declaration of the reward setting. Here we set the threshold to be 0.6 meter, keeping enough space between the robot and the obstacle in exploration. When the minimum depth was lower than the threshold or the robot location did not change for a period of time, we set the robot state to a termination and robot was reset to the start position. The reward for a termination is −50. At that time, we defined that a collision happened between the robot and an obstacle. Otherwise, if the robot kept moving, the reward was 1 to encourage moving. In the exploration procedure, the only target was to achieve obstacle avoidance, so the feedback of collision should be much larger than the normal moving. When updating the weights, if the reward was −50, that means the robot collide with an obstacle, and the target value for the state and action in this transition would also be −50. On the other hand, the target would be calculated by Bellman Equation if the robot state in this transition was keep moving. Algorithm 1 presents the whole framework of the control network training procedure. In every episode of the exploration, randomly choosing the command would increase the variety of the training batch. The $\epsilon$-greedy policy was implemented in the control network as well. The randomness would be less and less with the decreasing of $\epsilon$. Every time, after the execution of the chosen moving command, the new feature representations would be captured with rewarding 1, or the robot collided with the obstacle with rewarding −50. After storing the transition, the weights of Q-network were updated by the batch of transitions which were chosen randomly as well.

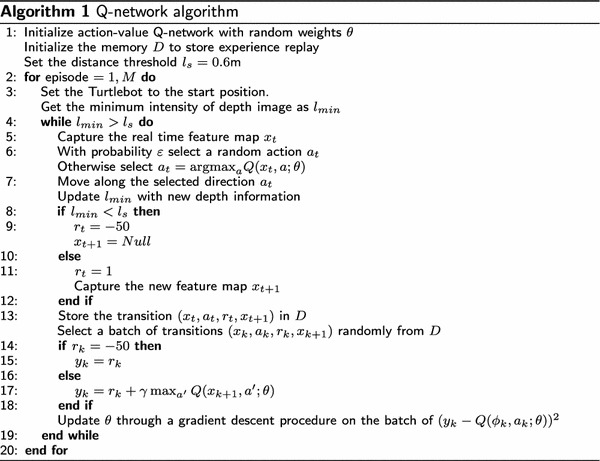



**Table 1:** Training parameters and their values

Parameter	Value
Batch size	32
Replay memory size	5000
Discount factor	0.85
Learning rate	0.000001
Gradient momentum	0.9
Max iteration	15,000
Step size	10,000
Gamma	0.1

**Table 2:** Setting of reward

State	Reward value
Collision or stop	−50
Keep moving	1

**Fig. 5:**
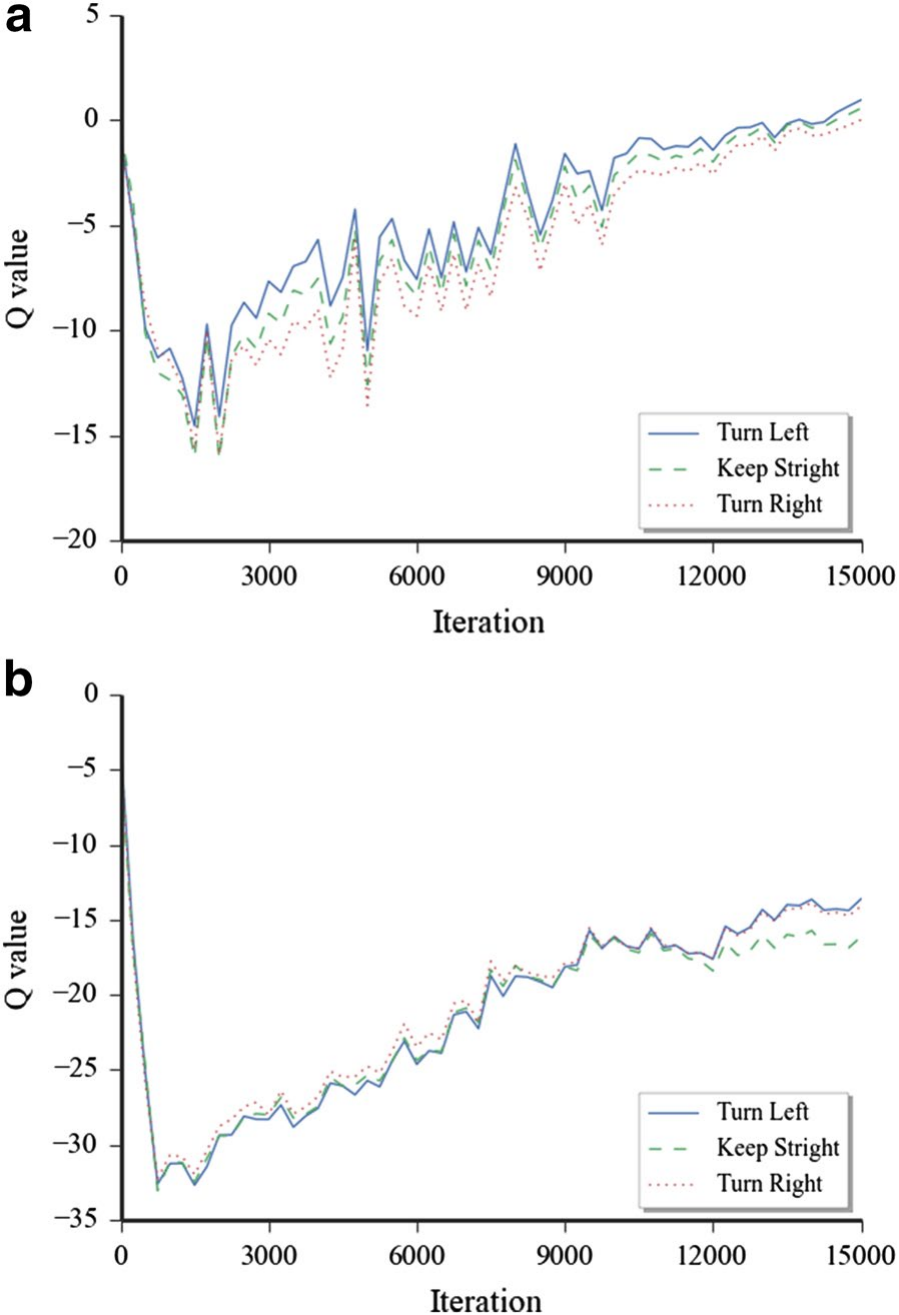
Q-value of the evaluation set calculated by the model at different iteration stages. **a** Q-value test for straight corridor, **b** Q-value test for circular corridor

**Fig. 6:**
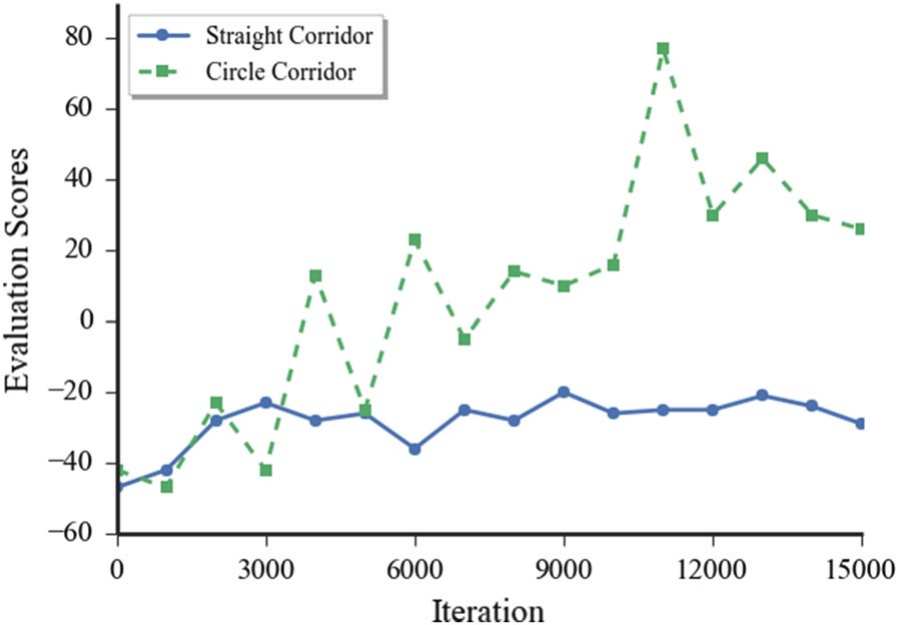
Average score of 5 times evaluation by using the trained model of every 1000 iteration steps

**Fig. 7:**
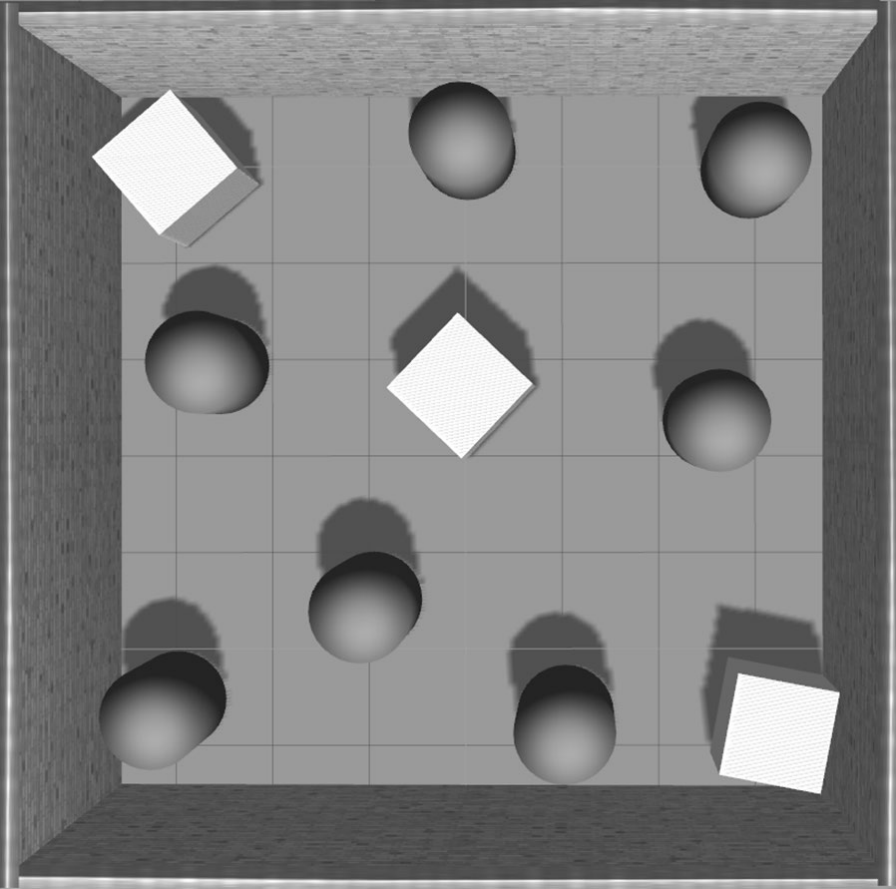
The more complicated environment for further experiment. But the proposed CNN-based reinforcement learning model is not converged to this new environment

### Environment design in Gazebo

Gazebo was used to build a simulation environment in this project. The robot used was a Turtlebot with two differential wheels, and a Kinect sensor was mounted on it. The whole project was implemented based on the interfaces of robot operation system (ROS). The system tracked the depth information from Turtlebot in Gazebo simulated environment. The command related to the highest Q-value in the control network would be transformed to the angel velocity for the Turtlebot by ROS topic.

## Results and discussion

Both of the feature learning in perception network and the updated of the control network weights were based on Caffe [22], a popular toolkit for deep learning. To evaluate the whole learning system, two different kinds of environments were designed as shown in Fig. 3. The first one consisted of a direct straight corridor. The other one consisted of a circularly connected corridor with more complicated depth information. Table 1 shows the training parameters and their values in gradient descent procedure of the control network learning implemented by Caffe. The step size means that the learning rate will multiply gamma after first 10,000 iterations, which means the learning rate of last 5000 iterations is 0.0000001.

### Training result

The control network learning system experienced 15,000 iterations in both of the two simulated environments.

Figure 4 shows the loss converging curves in the whole learning period. In every iteration step, the Euclidean loss between target Q-value and the predicted Q-value was calculated. The loss in Fig. 4 is the average value of the whole training batch in related iteration step. Because we chose the batch randomly in every step, the different training batch sets between the continues gradient descent steps led the apparent fluctuation in Fig. 4 in both of the environments. It also shows that the loss of both of the environments decreased rapidly in first 1500 iteration steps. And after that, the loss in the straight corridor was stable. But the convergence of the loss in the circular corridor environment was still decreasing apparently. That should be caused by the complexity of the depth information which needs more time to train the model.

### Test result

In the training process, the weights of the Q-network were saved regularly in every 300 steps of iteration. For both environments, we randomly chose several feature representations in different states as the test set, which correspond to different positions of the Turtlebot in the simulated world. Figure 5 shows the average Q-value of the test set for 3 different moving commands by using the trained weights of every 300 steps of the iteration.

It seems that all of the 3 target values converged toward a state with a certain value. The convergences of Q-value proved the stability of the control network system. Along with the shrinking of the learning rate after 10,000 steps of iteration, the fluctuation of the value was also reduced, especially for the straight corridor environment. The increment of the Q-value in the training procedure indicated that the control network was much more reliable with longer training time.

With the reward mentioned above, a direct feedback was the final scores the robot can achieve, with the trained strategy to choose the command related to the highest Q-value. The higher scores also meant that the Turtlebot would keep moving in the environment for a longer time and avoid more obstacles. But there was no apparent relation between the scores and the moving distance because the Turtlebot might move along a tortuous path. The width of the road in the simulated world was narrow enough that the robot cannot keep turning along the same direction rewarding positive infinite scores.

The trained model in every 1000 steps of iteration was tested 5 times in the related environment, and the result is shown in Fig. 6. It shows that the test scores of both environments increased at the first 2000 iterations. Scores tested in straight corridor world keep consistent near −20, which means that the robot moved 30 steps, with terminated reward −50. It was enough to arrive at the end of the straight corridor. The other one in the circular corridor environment was increased discontinuously. The highest test value in circular corridor environment was 80, which means that the robot moved 130 steps, with terminated reward −50. The Turtlebot should have finished a lap in that situation. We can imagine that the robot should keep moving forever along the circular corridor environment with infinite scores as a perfect model. But in the test, the robot will finally collide with obstacles every time. It is possible that the model is still not robust enough.

A more complicated simulated environment was also constructed as shown in Fig. 7. More obstacles were located to this simulated environment. However, no matter how long the control network was trained in this new environment, the exploration could not be accomplished. Even several more layers were added to the control network to improve the nonlinearity for complicated models, it sill could not converge. It is possible that the fixed pre-trained perception network limits the extension of the whole model. As mentioned before, the overfitting of the supervised learning model might not represent the new environment in Fig. 7 good enough.

## Conclusions

This paper presented a new approach to realize self-motivated exploration in an unknown environment by a CNN-based reinforcement learning network. The proposed modular network architecture provided a convenience way to transfer and update in the future. By separating the deep Q-network to a perception module and a control module, the exploration task was accomplished effectively. The test results in several simulated environments showed that the Turtlebot achieved obstacle avoidance ability, and it could travel freely in the simulated environment with the strategy learned by itself.

However, the failure in the more complicated environment also reflected the limitation of this paper. And several accessible aspects should be considered in the future to improve it. The whole DQN framework in real-time exploration should be implemented to train end-to-end. That means there should be no separated perception network for the feature representation. In the reinforcement learning procedure, the weights of the perception module should be updated at the same time. The perception ability of the system would be developed and adapted to the new environment naturally. Except for the depth information, the raw RGB image should also be considered as the input. With GPU accelerating, this should be also feasible.

The reward function should be redesigned as well because now every non-collision state was rewarded with the same score, which limits the interactions between the state and the robot in very few conditions. Finally, this model should be transformed to the application in real-world scenario.

## References

[1] Liu M, Colas F, Oth L, Siegwart R (2015). Incremental topological segmentation for semi-structured environments using discretized GVG. Auton Robots.

[2] Liu M, Colas F, Pomerleau F, Siegwart R. A Markov semi-supervised clustering approach and its application in topological map extraction. In: Intelligent robots and systems (IROS), 2012 IEEE/RSJ international conference on. IEEE; 2012. p. 4743–48.

[3] Lawrence S, Giles CL, Tsoi AC, Back AD (1997). Face recognition: a convolutional neural-network approach. Neural Netw IEEE Trans.

[4] Mnih V, Kavukcuoglu K, Silver D, Rusu AA, Veness J, Bellemare MG, Graves A, Riedmiller M, Fidjeland AK, Ostrovski G (2015). Human-level control through deep reinforcement learning. Nature.

[5] Tai L, Li S, Liu M. A deep-network solution towards model-less obstacle avoidance. In: Intelligent robots and systems (IROS), 2016 IEEE/RSJ international conference on. IEEE; 2016.

[6] Tai L, Liu M. A robot exploration strategy based on q-learning network. In: Real-time computing and robotics (RCAR), 2016 IEEE international conference on. IEEE; 2016

[7] Sutton RS, Barto AG (1998). Reinforcement learning: an introduction.

[8] Silver D, Huang A, Maddison CJ, Guez A, Sifre L, van den Driessche G, Schrittwieser J, Antonoglou I, Panneershelvam V, Lanctot M, Dieleman S, Grewe D, Nham J, Kalchbrenner N, Sutskever I, Lillicrap T, Leach M, Kavukcuoglu K, Graepel T, Hassabis D (2016). Mastering the game of go with deep neural networks and tree search. Nature.

[9] Kim HY, Jordan MI, Sastrys S. Ng AY. Autonomous Helicopter Flight via Reinforcement Learning. In: Thrun S, Saul SK, Schölkopf PB, editors. Advances in Neural Information Processing Systems. Cambridge, MA, USA: The MIT Press; 2004.

[10] Ng AY, Coates A, Diel M, Ganapathi V, Schulte J, Tse B, Berger E, Liang E. In: Ang MH, Khatib O, editors. Autonomous inverted helicopter flight via reinforcement learning. Experimental Robotics IX. Berlin: Springer; 2006. p. 363–72.

[11] Jamone L, Natale L, Nori F, Metta G, Sandini G (2012). Autonomous online learning of reaching behavior in a humanoid robot. Int J Humanoid Robot.

[12] He K, Zhang X, Ren S, Sun J. Deep residual learning for image recognition. arXiv preprint arXiv:1512.03385 (2015).

[13] Krizhevsky A, Sutskever I, Hinton GE. Imagenet classification with deep convolutional neural networks. In: Pereira F, Burges CJC, Bottou L, Weinberger KQ, editors. Advances in neural information processing systems; 2012. p. 1097–105. http://papers.nips.cc/book/advances-in-neural-information-processing-systems-25-2012.

[14] Li S, Huang H, Zhang Y, Liu M. A fast multi-scale convolutional neural network for object recognition. In: Real-time computing and robotics (RCAR), 2015 IEEE international conference on. IEEE; 2015.

[15] Sun Y, Wang X, Tang X. Deep convolutional network cascade for facial point detection. In: Proceedings of the IEEE conference on computer vision and pattern recognition; 2013. p. 3476–83

[16] Chen H, Wang P, Liu M. From co-saliency detection to object co-segmentation: a unified multi-stage low-rank matrix recovery approach. In: Robotics and biomimetics (ROBIO), 2015 IEEE international conference on. IEEE; 2015.

[17] Liu M, Scaramuzza D, Pradalier C, Siegwart R, Chen Q. Scene recognition with omnidirectional vision for topological map using lightweight adaptive descriptors. In: Intelligent robots and systems, 2009. IROS 2009. IEEE/RSJ international conference on. IEEE; 2009, p. 116–121.

[18] Liu M, Siegwart R (2014). Topological mapping and scene recognition with lightweight color descriptors for an omnidirectional camera. Robot. IEEE Trans..

[19] Liu M, Alper BT, Siegwart R. An adaptive descriptor for uncalibrated omnidirectional images—towards scene reconstruction by trifocal tensor. In: IEEE international conference on robotics and automation, 2013; 2013.

[20] Giusti A, Guzzi J, Ciresan D, He FL, Rodriguez JP, Fontana F, Faessler M, Forster C, Schmidhuber J, Di Caro G (2015). A machine learning approach to visual perception of forest trails for mobile robots. Robot Autom Lett IEEE.

[21] Mnih V, Kavukcuoglu K, Silver D, Graves A, Antonoglou I, Wierstra D, Riedmiller M. Playing atari with deep reinforcement learning. arXiv preprint arXiv:1312.5602 (2013).

[22] Jia Y, Shelhamer E, Donahue J, Karayev S, Long J, Girshick R, Guadarrama S, Darrell T. Caffe: Convolutional architecture for fast feature embedding. arXiv preprint arXiv:1408.5093 (2014).

